# Annual sulfur cycle in a warm monomictic lake with sub-millimolar sulfate concentrations

**DOI:** 10.1186/s12932-015-0021-5

**Published:** 2015-07-02

**Authors:** Nadav Knossow, Barak Blonder, Werner Eckert, Alexandra V Turchyn, Gilad Antler, Alexey Kamyshny

**Affiliations:** Department of Geological and Environmental Sciences, Faculty of Natural Sciences, Ben-Gurion University of the Negev, P.O. Box 653, 84105 Beer Sheva, Israel; Israel Oceanographic & Limnological Research Ltd., Yigal Allon Kinneret Limnological Laboratory, P.O. Box 447, Migdal, 14950 Israel; Department of Earth Sciences, University of Cambridge, Cambridge, CB2 3EQ UK

## Abstract

**Background:**

We studied the annual variability of the concentration and isotopic composition of main sulfur species and sulfide oxidation intermediates in the water column of monomictic fresh-water Lake Kinneret. Sulfate concentrations in the lake are <1 mM and similar to concentrations that are proposed to have existed in the Paleoproterozoic ocean. The main goal of this research was to explore biogeochemical constrains of sulfur cycling in the modern low-sulfate fresh-water lake and to identify which processes may be responsible for the isotopic composition of sulfur species in the Precambrian sedimentary rocks.

**Results:**

At the deepest point of the lake, the sulfate inventory decreases by more than 20% between March and December due to microbial sulfate reduction leading to the buildup of hydrogen sulfide. During the initial stages of stratification, sulfur isotope fractionation between sulfate and hydrogen sulfide is low (11.6 ‰) and sulfur oxyanions (e.g. thiosulfate and sulfite) are the main products of the incomplete oxidation of hydrogen sulfide. During the stratification and at the beginning of the lake mixing (July–December), the inventory of hydrogen sulfide as well as of sulfide oxidation intermediates in the water column increases and is accompanied by an increase in sulfur isotope fractionation to 30 ± 4 ‰ in October. During the period of erosion of the chemocline, zero-valent sulfur prevails over sulfur oxyanions. In the terminal period of the mixing of the water column (January), the concentration of hydrogen sulfide decreases, the inventory of sulfide oxidation intermediates increases, and sulfur isotope fractionation decreases to 20 ± 2 ‰.

**Conclusions:**

Sulfide oxidation intermediates are present in the water column of Lake Kinneret at all stages of stratification with significant increase during the mixing of the water column. Hydrogen sulfide inventory in the water column increases from March to December, and sharply decreases during the lake mixis in January. Sulfur isotope fractionation between sulfate and hydrogen sulfide as well as concentrations of sulfide oxidation intermediates can be explained either by microbial sulfate reduction alone or by microbial sulfate reduction combined with microbial disproportionation of sulfide oxidation intermediates. Our study of sulfur cycle in Lake Kinneret may be useful for understanding the range of biogeochemical processes in low sulfate oceans over Earth history.

## Introduction

In oxygen-depleted aquatic systems, sulfate is usually the most abundant water soluble electron acceptor for anaerobic microorganisms (Eq. 1). Oxygen depletion can result from water column stratification, particularly in limnic systems. Stratified water bodies with oxygen depletion in the deep water layer include lakes [1–5], ocean upwelling areas [6], marine regions with restricted water circulation (e.g. deep basins—[7]), fjords [8], and terminal seas [9, 10].

1 $$ 2\left({CH_{2} O} \right) + SO_{4}^{2 -} \to H_{2} S + 2HCO_{3}^{-} $$

In various aquatic and sedimentary systems a significant part of sulfide produced during microbial sulfate reduction can be re-oxidized either microbially or abiotically [11], rather than buried as pyrite. Sulfide oxidation can proceed via a variety of electron acceptors such as oxygen [12, 13], iron(III) (hydr)oxides [14, 15], manganese(III) complexes [16–18], manganese (IV) oxides [19] and nitrate [13]. Sulfide oxidation may result in the formation of sulfate (complete reoxidation), or sulfide oxidation intermediates such as polysulfides (S_n_^2−^), elemental sulfur (S^0^), thiosulfate (S_2_O_3_^2−^), polythionates (S_n_O_6_^2−^), and sulfite (SO_3_^2−^) [12, 15, 20–23].

In addition to the above mentioned reactions, iodate (IO_3_^−^), is the most common form of iodine in oxic water [24, 25]. In euphotic oxic ocean waters, significant fraction of inorganic iodine is found in the form of iodide, which is thermodynamically unstable [26]. Concentration of iodide in euphotic oxic waters is controlled by microbial reduction of iodate [27–29], photochemical decomposition of dissolved organic iodine [30, 31] as well as by oxidation of iodide to iodate [24, 32, 33]. In suboxic waters iodide becomes more stable than iodate [25]. In anoxic hydrogen sulfide rich waters iodide is the only detectable inorganic iodine species [26, 34] due to the fast reduction of iodate to iodide by hydrogen sulfide.

Iodate can oxidize hydrogen sulfide (Eqs. 2, 3) and its oxidation intermediates (Eqs. 4, 5) at the oxic-anoxic water interface [35–37]. The oxidation can either proceed to sulfate (Eqs. 2, 4), or sulfide oxidation intermediates may be formed (Eqs. 3, 5) [35, 36].

2 $$ 4IO_{3}^{-} + 3H_{2} S \to 4I^{-} + 3SO_{4}^{2 -} + 6H^{+} $$

3 $$ 2IO_{3}^{-} + 5H_{2} S + 2H^{+} \to I_{2} + 5S + 6H_{2} O $$

4 $$ IO_{3}^{-} + 3SO_{3}^{2 -} \to I^{-} + 3SO_{4}^{2 -} $$

5 $$ 2IO_{3}^{-} + 3S_{2} O_{3}^{2 -} + 3H_{2} O \to 2I^{-} + 6HSO_{3}^{-} $$

Some of the sulfide oxidation intermediates produced by sulfide oxidation are known to be involved in microbially mediated disproportionation, which does not always require an external electron donor or electron acceptor. This microbially-mediated disproportionation can utilize elemental sulfur [38–40], thiosulfate and sulfite [38, 41–43] to simultaneously produce sulfate and sulfide (Eqs. 6–8) [11].

6 $$ 4H_{2} O + 4S^{0} \to 3H_{2} S + SO_{4}^{2 -} + 2H^{+} $$

7 $$ 4SO_{3}^{2 -} + 2H^{+} \to H_{2} S + 3SO_{4}^{2 -} $$

8 $$ S_{2} O_{3}^{2 -} + H_{2} O \to H_{2} S + SO_{4}^{2 -} $$

Another sulfide oxidation intermediate that has been detected in the water columns of stratified lakes [44] and anoxic sediments [45] is thiocyanate (SCN^−^). Thiocyanate is formed by chemical reactions between free or metallo-complexed cyanide with reduced sulfur species, characterized by a sulfur–sulfur bond. Polysulfides and tetrathionate react fast with cyanide, and thiosulfate reacts more slowly by orders of magnitude [46–48].

**Figure 1:**
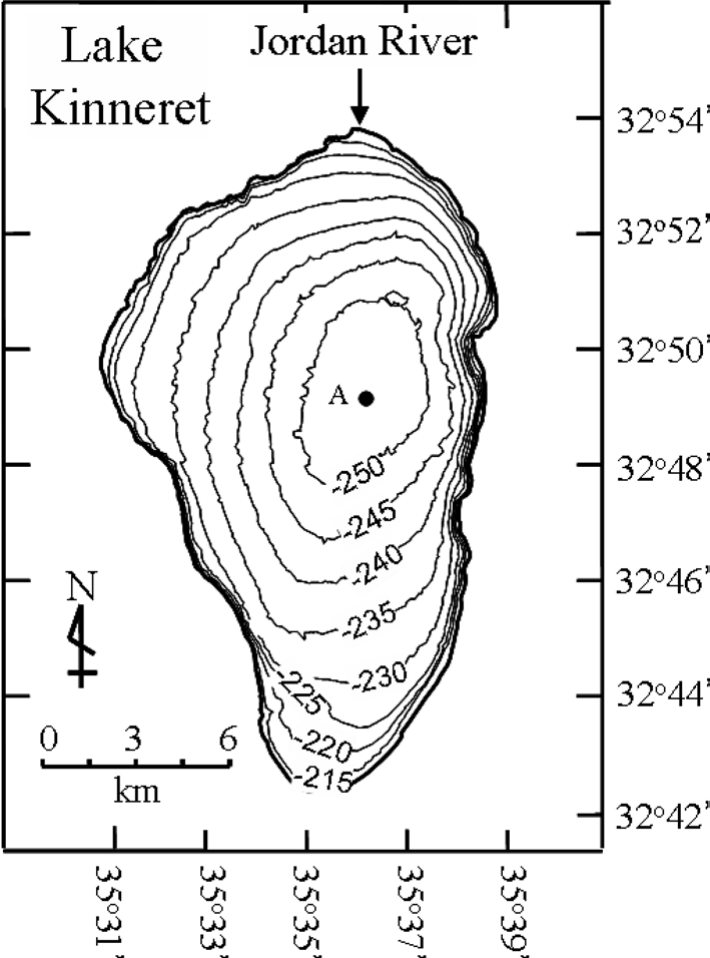
Map of Lake Kinneret, bathymetry, and the position of sampling station A.

In this publication we present an investigation that focuses on sulfur cycling in the water column of Lake Kinneret (Figure 1). The first reason for focusing the research on Lake Kinneret is that sub-millimolar sulfate concentrations and the presence of hydrogen sulfide in its hypolimnion make it a possible modern analog of the Proterozoic ocean. The other reason for choosing Lake Kinneret as a research system is the variation of its sulfur chemistry and ecology with seasonal timescales. This site provides a unique opportunity to study the cycling of sulfur as it relates to seasonal dynamics and to changes in the nature of sulfide oxidation pathways.

Lake Kinneret is situated in the northern part of the Afro-Syrian-Rift at 32°N latitude. The lake is 22 km long and 12 km wide at the widest point (with a surface area of 168 km^2^); the maximum and average depths are 42 and 24 m, respectively [1]. Lake Kinneret is an important drinking water source for Israel.

Thermal stratification of Lake Kinneret starts in April–May. The surface water temperature reaches a maximum of approximately 30°C in August, while the hypolimnion temperature is 15–16°C. The sulfate concentrations in the epilimnion of Lake Kinneret are around 600 μM, similar to the lower range of estimates for Proterozoic oceanic sulfate [49, 50]. Sulfate in the hypolimnion of Lake Kinneret can be depleted to concentrations below 200 μM, the upper limit of suggested Archean ocean sulfate concentrations [51]. The total sulfide level in the hypolimnion reaches 550 µM [52–54]. Microbial sulfate reduction has been identified as the dominant heterotrophic process in the hypolimnion of Lake Kinneret and responsible for the decomposition of 40% of the settling organic carbon from phytoplankton spring blooms [52].

Blooms of the brown phototrophic sulfur bacterium *Clorobium phaeobacteroides* are repeatedly observed from July until September in the metalimnion of the lake, at times when the chemocline rises into the photic zone [55]. In the presence of light, phototrophic bacteria efficiently oxidize metalimnetic sulfide under anoxic conditions [56]. High rates of chemolitotrophic primary production related to the oxidation of sulfide with oxygen as electron acceptor and chemical sulfide oxidation are also observed from November to January during the gradual destratification process when sulfidic hypolimnetic and oxic epilimnetic water are mixed [57]. Malodorous volatile organic sulfur compounds (e.g. dimethyl sulfide, dimethyl disulfide, dimethyl trisulfide, and dimethyl tetrasulfide) were detected in Lake Kinneret water column during the algal blooms. Inorganic polysulfides were shown to be precursors of these compounds in the lake [58, 59].

The chemical and isotope composition of the various products formed by sulfate reducing, sulfur disproportionating, and sulfide oxidizing bacteria in modern aquatic systems that have conditions similar to those believed to have existed in ancient oceans, are usually used for comparison with chemical and isotopic composition of ancient sedimentary rocks [60–63]. Lake Kinneret represents a modern analog of a global ocean in the Proterozoic. This period of the Earth’s history was characterized by a rise of oxygen concentration in the atmosphere [64], followed by rise of sulfate concentrations in the ocean from 80 to 200 µM during the Archean [51, 65] to 0.5–2.4 mM during the mid-Proterozoic [49] and the evolution of a ferruginous deep ocean to a “Canfield Ocean” with sulfidic deep water [66]. Recent work has suggested that this picture may be complicated with transient high concentrations of sulfate [67] and possible ferruginous conditions in the latest Neoproterozoic. However, the overall picture of the Proterozoic involves low sulfate concentrations in the majority of the ocean. At low sulfate concentration (<1 mM), sulfur isotope fractionation between sulfate and sulfide associated with freshwater microbial sulfate reduction decreases to around 7–30 ‰ [51], and even as low as −3 ‰ when sulfate concentrations are as low as 10 µM [68]. This phenomenon was used to calibrate sulfate concentrations in the Archean ocean [51]. The δ^34^S values of sedimentary sulfides differed not more than 10 ‰ from the mantle value of 0 ‰ [69 and references therein]. The difference in the δ^34^S values of sulfide and sulfate during this period was usually <15 ‰ [69, 70], with the highest value reaching 21 ‰ [71]. The low sulfur isotopic fractionation is interpreted as indication for sulfate concentrations <200 µM. The rise of marine sulfate concentrations in the Proterozoic was accompanied by an increase in sulfur isotope fractionation [69 and references therein].

On the other hand, various microbial processes such as sulfide oxidation and subsequent disproportionation of zero-valent sulfur are known to increase sulfur isotope fractionation between sulfate and sulfide [40]. Microbial sulfur disproportionation is known to co-exist with microbial sulfate reduction for at least 3.49–3.50 billion years [72, 73]. These biogeochemical processes are reflected in the chemical and isotopic compositions of sedimentary rocks.

In this study, we determined not only concentrations of various sulfur species but also the isotopic composition (δ^34^S) of sulfate (δ^34^S_SO4_), hydrogen sulfide (δ^34^S_H2S_), and zero-valent sulfur (δ^34^S_So_) in the water column of Lake Kinneret. We coupled the analyses of sulfur isotopes of the various sulfur species with that of the oxygen isotopes in aqueous sulfate. The δ^18^O_SO4_ is affected by microbial-driven sulfur transformations (e.g. microbial sulfate reduction, disproportionation, and sulfide oxidation), but in a different manner to the sulfur isotope fractionation, and thus yields different and unique insight into sulfur cycling in an aquatic system. During microbial sulfate reduction, the δ^34^S_SO4_ of the residual sulfate typically increases while sulfate is consumed. At the same time the δ^18^O_SO4_ may initially increase but ultimately reaches isotope equilibrium with coexisting water [74–80]. During initiation of microbial sulfate reduction, the increase in both the sulfur and oxygen isotopes in the residual sulfate may yield insight into the establishment of the annual sulfur cycle within the lake [77, 78, 81–83].

The main goal of this research was to investigate the time–space variability of the concentration and isotopic compositions of main and intermediate sulfur species during the annual cycle of Lake Kinneret, a freshwater lake with sulfate concentrations similar to those in the Proterozoic ocean. Specifically, our aim was to understand how sulfur cycling in the lake is affected by geochemical conditions similar to the Proterozoic ocean (e.g. low sulfate concentrations), and geochemical parameters, which make Lake Kinneret different from the Proterozoic ocean (e.g. high oxygen concentrations in the epilimnion and annual cycle of hydrographic conditions). This is important in order to constrain the factors that led to relatively low sulfur isotope fractionation in Proterozoic sedimentary rocks.

## Results

### Annual thermal and chemical stratification pattern of Lake Kinneret

Six sampling cruises were undertaken on-board the R/V Hermona of the Yigal Allon Kinneret Limnology Laboratory. The first sampling (LK1) was performed on March 12th during the water column mixis, the second sampling (LK2) on May 14th during the initial period of thermal stratification of Lake Kinneret, the third sampling (LK3) on July 5th during the period characterized by the shallowest chemocline position, the fourth sampling (LK4) was performed on October 13th, during stable chemocline conditions, the fifth sampling (LK5) on December 12th, 2012 at the beginning of the erosion of the chemocline, and the sixth sampling (LK6) on January 17th, 2013 during the last stage of the erosion of the chemocline (Table 1).

**Table 1:** Sampling dates and water depths of the chemocline

Sampling	Date	Chemocline depth (m)
LK1	06/03/2012	No chemocline
LK2	14/05/2012	33.1
LK3	05/07/2012	11.5
LK4	31/10/2012	18.5
LK5	12/12/2012	20.5
LK6	17/01/2013	38.0

Chemocline depth was defined as the shallowest depth with hydrogen sulfide concentration above 1 µM.

**Figure 2:**
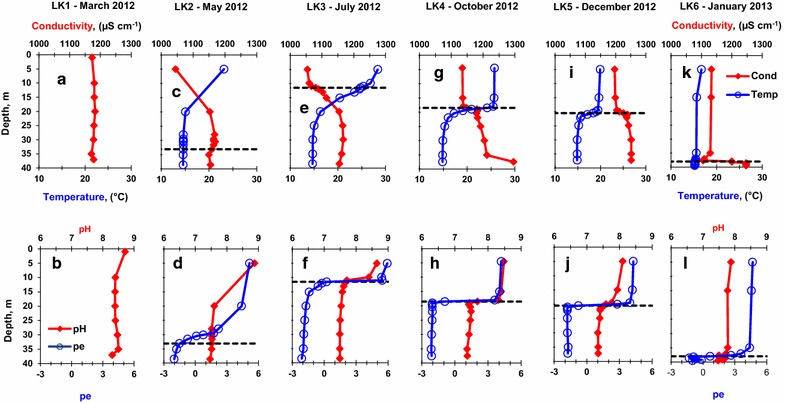
Seasonal variations in the depth profiles of conductivity, temperature, pH, and pe in Lake Kinneret. *Black dashed lines* denote the chemocline depth. Chemocline depth was defined as the shallowest depth with hydrogen sulfide concentration above 1 μM. **a** Conductivity during the LK1 sampling,** b** pH during the LK1 sampling,** c** conductivity and temperature during the LK2 sampling,** d** pH and pe during the LK2 sampling,** e** conductivity and during the LK3 sampling,** f** pH and pe during the LK3 sampling,** g** conductivity and temperature during the LK4 sampling,** h** pH and pe during the LK4 sampling,** i** conductivity and temperature during the LK5 sampling,** j** pH and pe during the LK5 sampling,** k** conductivity and temperature during the LK6 sampling,** l** pH and pe during the LK6 sampling.

LK1 sampling (Figure 2a) conductivity profile was typical of a mixis period and was characterized by a uniform conductivity of about 1,180 µS cm^−1^ throughout the water column. The temperature was not measured during this sampling. LK1 sampling (Figure 2b) pH profile was relatively uniform with a pH of 8.7 at a depth of 1.0 m and a pH of 8.3 at the bottom (37.0 m); pe was not measured during this sampling. During the LK2 sampling (Figure 2c), which represents the initial stage of stratification, conductivity profile was characterized by a gradual increase with depth down to 30.5 m followed by a slight decrease below the chemocline. The temperature decreased with depth from 23.1 at the surface to 14.6°C near the bottom. The pH (Figure 2d) decreased from 8.9 to 7.6 with depth while the pe decreased from 5.2 at the surface to −2.0 in the bottom waters. At the LK3, LK4 and LK5 samplings, which represent a period of stable stratification of the water column (Figure 2e, g, i), the lowest conductivity was detected at the lake surface. At the chemocline, the conductivity rapidly increased and stabilized deeper in the hypolimnion. While the highest water temperature was measured at the surface during this time period, the temperature sharply decreased in the chemocline and stabilized at 15°C in the hypolimnion. The pH and pe of the LK3, LK4 and LK5 profiles (Figure 2f, h, j) both slightly decreased with depth in the epilimnion, sharply dropped at the chemocline, and gradually decreased with depth in the hypolimnion before stabilizing at similar values in the deep waters. During the LK6 sampling, which was performed several days before the full mixing of the lake (Figure 2k), the conductivity was 1,130 µS cm^−1^ in most of the epilimnion, decreased to a minimum of 1,106 µS cm^−1^ near the chemocline (35.0–38.0 m), and increased rapidly with depth to a maximum of 1,247 µS cm^−1^ near the bottom of the lake (39.5 m). The temperature was rather uniform between 16.7°C at the surface and 15.2°C at the bottom. The pH was constant around 7.8–7.9 in the epilimnion (Figure 2l) and dropped sharply at the chemocline down to a minimum of 7.48 at the bottom of the lake. The pe profile had a maximum of 4.7 at the surface that remained constant to a depth of 35.0 m and then sharply decreased to −1.0 just above the bottom.

### Iodide

**Figure 3:**
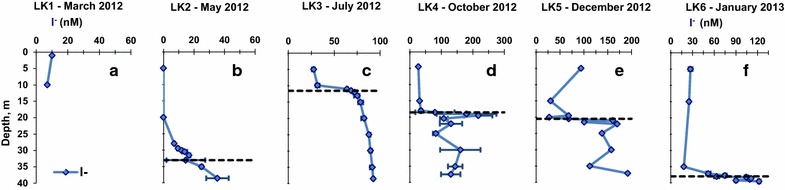
Seasonal variations in the depth profiles of iodide in Lake Kinneret.* Black dashed lines* denote the chemocline depth.* Error bars* represent the standard deviation on duplicate samples.** a** LK1 sampling,** b** LK2 sampling,** c** LK3 sampling,** d** LK4 sampling,** e** LK5 sampling,** f** LK6 sampling.

During the LK1 sampling, iodide concentrations were measured only at two depths (Figure 3a) and were 7–10 nM. LK2 sampling profile of iodide (Figure 3b) showed an increase of concentration with depth, which was especially pronounced below the chemocline (33.1 m). The maximum concentration of I^−^, 35.0 ± 7.0 nM, was detected just above the bottom of the lake (38.6 m). During the LK3 sampling (Figure 3c), iodide concentration was 28.0 ± 1.0 nM at 5 m depth, and a sharp increase in concentration to 64.0 ± 1.0 nM was detected just above the chemocline (11.5 m). In the hypolimnion, the concentration of iodide rose slowly with depth to a maximum of 92.0 ± 1.0 nM just above the bottom of the lake (38.5 m). Iodide concentrations during the LK4 sampling (Figure 3d) were stable at 27.0–35.0 nM in the epilimnion, and had a sharp maximum of 217 ± 45 nM just below the chemocline. In the hypolimnion iodide concentration decreased to 82–160 nM range. Iodide concentration during the LK5 sampling (Figure 3e) had an unusually high concentration, 94.0 nM, at 5 m depth, decreased to 27.0 nM just above the chemocline (20.5 m), and rose sharply to 170 nM at a depth of 22.1 m just below the chemocline. In the hypolimnion, iodide concentration remained in the 101–192 nM range. During the LK6 sampling (Figure 3f), iodide concentration in the epilimnion was 18–27 nM with a sharp rise, just below the chemocline to 122 ± 2 nM at the bottom of the lake (37.2 m).

### Oxygen

**Figure 4:**
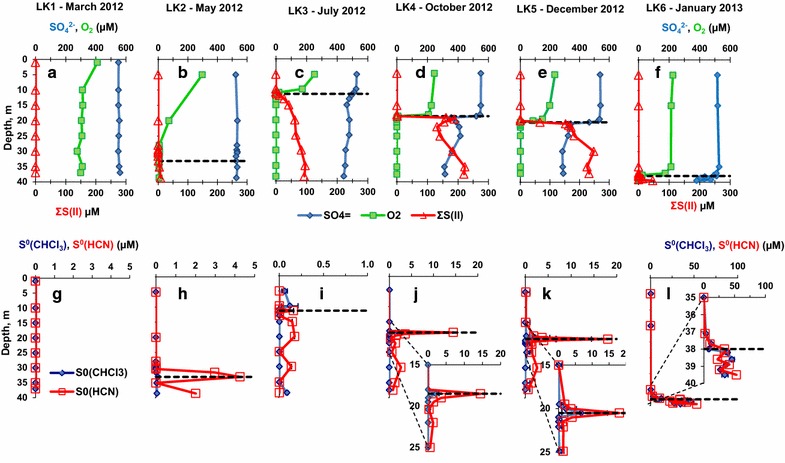
Seasonal variations in the depth profiles of dissolved oxygen, sulfate, total S(II), chloroform extractable zero-valent sulfur, and hydrogen cyanide-reactive zero-valent sulfur in Lake Kinneret.* Black dashed lines* denote the chemocline depth. In panels** a**–**f** *error bars* are smaller than the symbols.** a** Dissolved oxygen, sulfate, and total S(II) during the LK1 sampling,** b** dissolved oxygen, sulfate, and total S(II) during the LK2 sampling,** c** dissolved oxygen, sulfate, and total S(II) during the LK3 sampling,** d** dissolved oxygen, sulfate, and total S(II) during the LK4 sampling,** e** dissolved oxygen, sulfate, and total S(II) during the LK5 sampling,** f** dissolved oxygen, sulfate, and total S(II) during the LK6 sampling,** g** chloroform extractable zero-valent sulfur and hydrogen cyanide-reactive zero-valent sulfur during the LK1 sampling,** h** chloroform extractable zero-valent sulfur and hydrogen cyanide-reactive zero-valent sulfur during the LK2 sampling,** i** chloroform extractable zero-valent sulfur and hydrogen cyanide-reactive zero-valent sulfur during the LK3 sampling,** j** chloroform extractable zero-valent sulfur and hydrogen cyanide-reactive zero-valent sulfur during the LK4 sampling,** k** chloroform extractable zero-valent sulfur and hydrogen cyanide-reactive zero-valent sulfur during the LK5 sampling,** l** chloroform extractable zero-valent sulfur and hydrogen cyanide-reactive zero-valent sulfur during the LK6 sampling.

**Figure 5:**
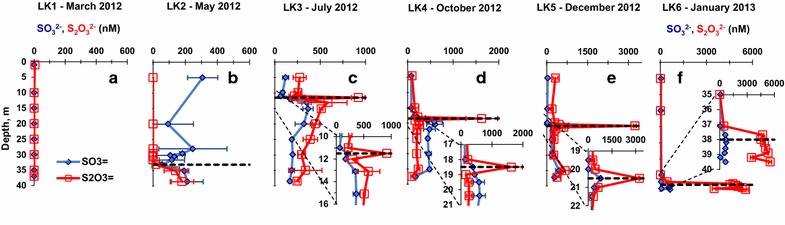
Seasonal variations in the depth profiles of sulfite, thiosulfate in Lake Kinneret. *Black dashed lines* denote the chemocline depth.** a** LK1 sampling,** B** LK2 sampling,** c** LK3 sampling,** d** LK4 sampling,** e** LK5 sampling,** f** LK6 sampling. Please notice enlarged profile sections on panels** c**–**f**.

During the LK1 sampling (Figure 4a), dissolved oxygen concentrations were 411 µM at a depth of 1.0 m, decreased to 312 µM at 10.0 m depth, and showed no depth dependence below that depth. The LK2 sampling dissolved oxygen profile (Figure 4b) showed a maximum of 298 µM at 5 m depth that decreased rapidly to 6.9 µM at a depth of 28.0 m, then further decreased to a minimum of 5.7 µM at a depth of 35.1 m and increased slightly to 6.4 µM just above the bottom. During the LK3, LK4, LK5 and LK6 samplings (Figure 5c–f) dissolved oxygen concentration sharply dropped from saturation in the epilimnion to below minimum detection limit (MDL) just above the chemocline and remained below the MDL in the hypolimnion.

### Dissolved iron

While dissolved iron concentrations were not measured during LK1 and LK2 sampling periods, they were below the MDL (1 µM) at all depths except for 2.2 µM at the bottom (38.5 m) and 3.0 µM at the chemocline (18.5 m), respectively during the LK3 and LK4 samplings (data not shown). During the LK6 sampling, the dissolved iron concentration was below the MDL above 38 m depth then rose to 1.1 µM at the chemocline (38.0 m) and then further to a maximum of 1.6 µM at a depth of 38.9 m (not shown). These results agree with the data previously published by Shaked et al. [84], who showed that total iron concentration in the water column of the lake is always below 1.2 µM.

### Total sulfur(II) and sulfate

During the LK1 sampling (Figure 4a), total S(II) concentrations were below the MDL throughout the well oxygenated water column while sulfate concentrations were in the 550–561 µM range at all depths. During the LK2 sampling (Figure 4b), the total S(II) concentrations were below the MDL between 5.0 and 31.6 m depths but rose to 0.8 ± 0.2 µM at the chemocline (33.1 m) and further to 8.0 ± 0.1 µM at the deepest sampled horizon (38.6 m). Sulfate concentrations remained in the 524–535 µM range throughout the water column. Total S(II) concentrations during the LK3 sampling were below the MDL (Figure 4c) in the epilimnion but increased to 2.1 ± 0.2 µM and further to 92.9–95.8 µM at 35–38.5 m depth. Sulfate concentration dropped from 550 ± 2 µM and 487 ± 3 µM between 5.0 and 13.0 m depths, followed by a further decrease to 443 ± 4 µM in the bottom waters of the lake (38.5 m). During the LK4 sampling (Figure 4d), total S(II) concentrations were below the MDL in the epilimnion. S(II) concentrations rose to 1.6 ± 0.2 µM at 18.5 m depth and further increased with depth to 92.9 ± 8.0 µM at the deepest sampled horizon (37.5 m). Sulfate concentrations in the epilimnion were 550–545 µM and dropped to a local minimum of 346 ± 3 µM at a depth of 19.5 m, then remained constant in the 315–416 µM range. Total S(II) concentrations during the LK5 sampling were first detectable (Figure 4e) at a depth of 20.5 m (66.9 ± 3.0 µM) and continued to increase with depth to 248 ± 11 µM at 30 m. Below this depth total S(II) concentrations were uniform. Sulfate concentrations in the epilimnion were 527–539 µM and sharply dropped just below the chemocline (20.5 m) to 334 ± 0 µM at a depth of 22.1 m with a further decrease with depth to a minimum of 232 ± 3 µM just above the bottom of the lake (37.2 m). During the LK6 sampling (Figure 4f), total S(II) concentrations were below the MDL between 5.0 and 37.7 m depth. In the chemocline, which was situated at 38.0 m depth, total S(II) was 3.3 ± 0.1 µM and rose to a maximum of 109 µM just above the bottom. The concentration of sulfate was 517 ± 1 µM at 5 m depth, decreased slightly to 495 ± 1 µM above the chemocline at a depth of 37.7 m, and then dropped sharply to 407 ± 2 µM at a depth of 38.6 m. The concentration of sulfate at the deepest sampled horizon, 39.8 m, was 333 ± 5 µM.

## Chloroform-extractable sulfur $\left({{\rm S}_{{{\text{CHCl}}3}}^{0}} \right)$ and hydrogen cyanide-reactive sulfur $\left({{\rm S}_{\text{HCN}}^{0}} \right)$

Zero-valent sulfur concentrations analyzed by both techniques were below the MDL in the entire water column during the LK1 sampling period (Figure 4g). While $\left({{\rm S}_{{{\text{CHCl}}3}}^{0}} \right)$ concentrations were below the MDL throughout the entire water column during the LK2 sampling period (Figure 4h), $\left({{\rm S}_{\text{HCN}}^{0}} \right)$ concentrations were below the MDL above 31.6 m then increased to peak at the chemocline (4.26 µM at 33.1 m) before rebounding to 2.02 µM in the deepest sampled horizon (38.6 m). During the LK3 sampling (Figure 4i), $\left({{\rm S}_{{{\text{CHCl}}3}}^{0}} \right)$ concentration was above 100 nM only at the water depths of 9.9 m. while $\left({{\rm S}_{\text{HCN}}^{0}} \right)$ was above the detection limit at 11.5 m, 15–20 m and 29.9 m depths. During the LK4 sampling (Figure 4j), $\left({{\rm S}_{{{\text{CHCl}}3}}^{0}} \right)$ was below the MDL throughout the entire water column except for one point in the chemocline (18.5 m) with a concentration of 2.18 ± 0.83 µM. $\left({{\rm S}_{\text{HCN}}^{0}} \right)$ concentrations in the epilimnion were below the MDL, increased sharply to 14.47 µM at the chemocline, and then dropped to 0.82 µM at the deepest sampled horizon (37.5 m). During the LK5 sampling (Figure 4k), $\left({{\rm S}_{{{\text{CHCl}}3}}^{0}} \right)$ was below the MDL throughout the entire water column except for one point at the chemocline depth (20.5 m) with a concentration of 6.75 ± 0.76 µM. $\left({{\rm S}_{\text{HCN}}^{0}} \right)$ surface concentrations were 0.14 µM at 5 m depth, increased sharply to 18.1 µM at the chemocline, then decreased to 4.0 µM at a depth of 21 m and down to 0.58 µM at the deepest sampled horizon (37.2 m). During the LK6 sampling (Figure 4l), $\left({{\rm S}_{{{\text{CHCl}}3}}^{0}} \right)$ concentration was below the MDL in the epilimnion. It increased to a local maximum of 12.24 ± 0.23 µM at a depth of 37.7 m, and to a maximum of 45.5 ± 3.88 at a depth of 38.6 m. $\left({{\rm S}_{{{\text{CHCl}}3}}^{0}} \right)$ concentration then dropped to 34.14 ± 5.0 µM just above the bottom (39.5 m depth). $\left({{\rm S}_{\text{HCN}}^{0}} \right)$ concentrations were below MDL at 5 m depth and remained the same down to 35.0 m depth where they increased sharply to 33.5 µM at the chemocline (38.0 m) and further to a maximum of 52.6 µM just above the bottom (39.5 m depth).

### Sulfite and thiosulfate

Sulfite and thiosulfate concentrations were either low or below the MDL throughout the entire water column during the LK1 sampling (Figure 5a). During the LK2 sampling (Figure 5b), sulfite concentrations generally decreased from a maximum of 308 ± 94 nM at 5 m depth in the epilimnion to a minimum of 56 ± 22 nM at the depth of the chemocline (33.1 m) and then rebounded to 212 ± 99 nM at the deepest sampled horizon (38.6 m). Thiosulfate concentrations were below the MDL in the epilimnion and then rose in the hypolimnion to 179 ± 73 nM just above the bottom (38.6 m). During the LK3 sampling (Figure 5c), sulfite slightly decreased in concentration from the surface to its lowest concentration 65 ± 13 nM at 11.0 m depth, just above the chemocline, then increased sharply below the chemocline to its highest concentration 366 ± 57 nM at a depth of 15.1 m. This increase was followed by slight decrease with depth in the hypolimnion to 165 ± 17 nM just above the bottom (38.5 m). Thiosulfate had a similar profile with an epilimnion concentration 217–274 nM, an increase in concentration to a maximum of 920 ± 75 nM at the chemocline (11.5 m) followed by a progressive decrease to 244 ± 24 nM at the deepest depth sampled (38.5 m). During the LK4 sampling (Figure 5d), sulfite had a minimum concentration of 76 ± 3 nM at 4.5 m depth, it increased to a maximum of 601 ± 183 nM below the chemocline (20.4 m) and decreased to 171 ± 30 nM just above the bottom (37.5 m). The thiosulfate profile was similar to the sulfite profile with a minimum concentration of 95 ± 2 nM at 4.5 m depth, a sharp increase at the chemocline (18.5 m) to 1625 ± 35 nM followed by decrease to 93 ± 29 nM just above the bottom (37.5 m). During the LK5 sampling (Figure 5e), sulfite had a minimum concentration of 33 ± 17 nM at 5 m depth, increasing sharply at the chemocline (20.5 m) to 795 ± 45 nM. The concentration then decreased to 378 ± 31 nM at a depth of 21.0 m and rose back to a local maximum of 455 ± 130 nM at a depth of 35 m. Near the bottom of the lake (37.2 m) the sulfite concentration was 282 ± 57 nM. A constant thiosulfate concentration of 316 ± 92 nM, was detected in the epilimnion and increased sharply at the chemocline to form a peak at 3,242 ± 38 nM before stabilizing in the hypolimnion around 331–672 nM. During the LK6 sampling (Figure 5f), the lowest concentration of sulfite (45 ± 16 nM) was detected at 5 m depth. Sulfite concentrations rose sharply just below the chemocline (38 m) to a maximum of 710 ± 22 nM at a depth of 38.2 m and then dropped to 634 ± 13 nM just above the bottom at a 39.5 m depth. Thiosulfate concentration had a similar depth profile with a minimum concentration of 8 ± 1 nM at 5 m depth rising slowly to 437 ± 60 nM at a depth of 37.1 m and then more sharply to 4,725 ± 127 nM at 37.7 m. In the hypolimnion, the thiosulfate concentration increased more slowly to a maximum of 5,540 ± 177 nM just above the bottom (39.5 m).

### Sulfate δ^34^S

**Figure 6:**
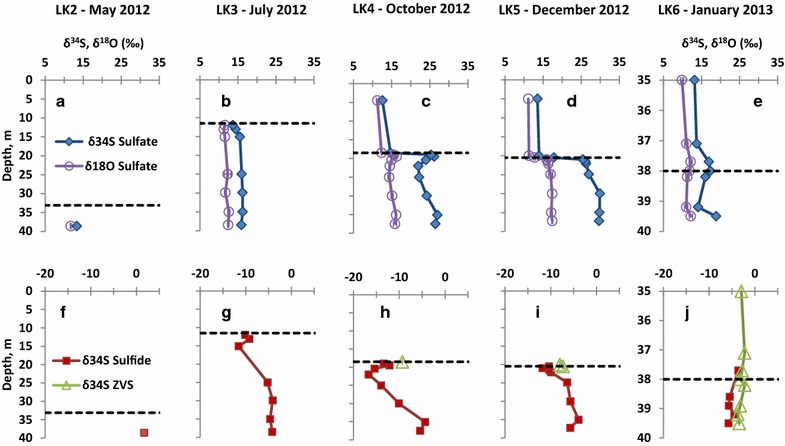
Seasonal variations in the depth profiles of the sulfur isotopic composition, δ^34^S, in sulfate, sulfide, and zero-valent sulfur, as well as of oxygen, δ^18^O, in sulfate in Lake Kinneret.* Black dashed lines* denote the chemocline depth.** a** Isotopic composition of sulfate during the LK2 sampling,** b** isotopic composition of sulfate during the LK3 sampling,** c** isotopic composition of sulfate during the LK4 sampling,** d** isotopic composition of sulfate during the LK5 sampling,** e** isotopic composition of sulfate during the LK6 sampling,** f** isotopic composition of sulfide during the LK2 sampling,** g** isotopic composition of sulfide during the LK3 sampling,** h** isotopic composition of sulfide and zero-valent sulfur during the LK4 sampling,** i** isotopic composition of sulfide and zero-valent sulfur during the LK5 sampling,** j** isotopic composition of sulfide and zero-valent sulfur during the LK6 sampling. Please notice different depth scale in panels** e** and** j**.* Error bars* are smaller than the symbols.

During the LK2 sampling (Figure 6a), only one depth was sampled for δ^34^S_SO4_ (38.6 m) which yielded a value of 13.3 ‰. During the LK3 sampling (Figure 6b), only the sulfidic bottom water was sampled, the δ^34^S_SO4_ of the chemocline (33.1 m) was around the same value, and the δ^34^S gradually increased with depth to a maximum of 16.2 ‰ at 29.9 m. During the LK4 sampling (Figure 6c), the δ^34^S_SO4_ slightly increased from 12.6 ‰ at the shallowest horizon sampled (4.5 m) to 14.7 ‰ at the chemocline (18.5 m). It rose steeply to a local maximum of 26.2 ‰ at a depth of 19.5 m before decreasing to a local minimum of 22.0 ‰ at a depth of 22.0 m. Eventually, the δ^34^S_SO4_ rebounded in the hypolimnion to reach a maximum value of 27.0 ‰ at a depth of 35.1 m and then slightly decreased to 26.5 ‰, at the deepest sampled horizon (37.5 m). During the LK5 sampling (Figure 6d), the profile of δ^34^S_SO4_ was similar to that of the LK4 sampling. The δ^34^S_SO4_ slightly increased from 13.5 ‰ at 5.0 m depth to 13.9 ‰ at 19.5 m, followed by a sharp increase with depth to 26.1 ‰ 1 m below the chemocline at a depth of 21.5 m. In contrast to the LK4 sampling, however, δ^34^S_SO4_ did not dip in the hypolimnion but continued to increase to its highest value (29.8 ‰) detected at 35.0 m depth. During the LK6 sampling (Figure 6e), the δ^34^S_SO4_ was 12.8 ‰ at 5 m depth (not shown) and it slowly increased to 13.8 ‰ at a depth of 37.1 m. A local maximum of 17.3 ‰ was measured at the chemocline depth (38 m) followed by a local minimum of 14.1 ‰ at 39.2 m. The maximum δ^34^S_SO4_, 18.9 ‰, was detected at the deepest sampled horizon (39.5 m).

### Sulfate δ^18^O

The shapes of δ^18^O_SO4_ depth profiles follow closely the δ^34^S_SO4_ profiles. These profiles are characterized by an increase in δ^18^O_SO4_ values at the chemocline. Therefore only minimum and maximum values will be noted here. During the LK2 sampling (Figure 6a), only one horizon was sampled at 38.5 m, and a δ^18^O_SO4_ of 11.8 ± 0.2 ‰ was measured in the hypolimnion. For LK3 sampling (Figure 6b), a minimum δ^18^O_SO4_ of 11.5 ± 0.4 ‰ was measured at a depth of 12.0 m and the highest δ^18^O _SO4_ 12.6 ± 0.3 ‰ was measured at 35.0 m. LK4 (Figure 6c) and LK5 samplings (Figure 6d) showed the same overall profiles with the lowest δ^18^O_SO4_ of 11.2 ± 0.1 ‰ and 11.1 ± 0.2 ‰ at depth of 15.0 and 5.0 m, respectively. The highest δ^18^O_SO4_ were 16.3 ± 0.2 ‰ and 17.4 ± 0.2 ‰ at depths of 19.5 and 37.2 m, respectively. Finally, LK6 sampling (Figure 6e) had a minimum δ^18^O_SO4_ of 9.9 ± 0.2 ‰ at a depth of 35.0 m and a maximum of 12.2 ± 0.1 ‰ at a depth of 19.5 m.

### Sulfide δ^34^S

Only one horizon was sampled for the δ^34^S_H2S_ during the LK2 sampling (Figure 6f), and the isotopic composition of the point (38.6 m) was 1.7 ‰. During the LK3 sampling (Figure 6g), sulfide concentrations were high enough to allow isotopic analysis below 11.5 m only. The δ^34^S_H2S_ increased from −11.0 ‰ at the chemocline to maximum of −9.2 ‰ at a depth of 13.1 m, and then decreased to −11.5 ‰ at 15.1 m before increasing again with depth to −4.1 to −4.7 ‰. During the LK4 sampling (Figure 6h), the δ^34^S_H2S_ was similar to the LK3 sampling profile. The δ^34^S_H2S_ was −14.3 ‰ at 18.5 m followed by a local maximum of −12.1 ‰ at 19.5 m, a minimum of −16.7  ‰ at 22.0 m and a maximum of −5.4  ‰ at 35.1 m. LK5 (Figure 6i) and LK6 sampling (Figure 6j) δ^34^S_H2S_ profiles showed similar trends. The shallowest horizons with concentration of sulfide high enough to allow isotope analysis were 20.0 and 38.0 m, and δ^34^S values were −10.4  ‰ and −3.6 ‰, respectively. During the LK5 sampling, a minimum of −11.8 ‰ at a depth of 21.0 m and a maximum of −3.9 ‰ at a depth of 35.0 m were detected. During the LK6 sampling, the profile had a local minimum of −5.7  ‰ at 38.9 m followed by a local maximum of −4.5  ‰ at 39.2 m.

### Zero-valent sulfur δ^34^S

During the LK1, LK2 and LK3 samplings, δ^34^S_zvs_ was not measured due to the low concentration of zero-valent sulfur (ZVS). During the LK4 sampling (Figure 6h), δ^34^S_ZVS_ was retrieved from one horizon at 18.5 m where δ^34^S_zvs_ was −9.4 ± 0.0 ‰. During the LK5 sampling (Figure 6i), δ^34^S_ZVS_ was retrieved from two horizons at 20.0 and 20.5 m, where δ^34^S_zvs_ were −8.0 ± 0.1 ‰ and −5.4 ± 0.1 ‰, respectively. During the LK6 sampling (Figure 6j), zero-valent sulfur concentrations were high enough to allow δ^34^S_zvs_ analysis from both the oxic and sulfidic waters. The δ^34^S_zvs_ was −2.2 ‰ to −3.4 ‰ at 35.0–39.5 m depths.

## Discussion

The sulfur cycle in the monomictic Lake Kinneret was studied over 1 year cycle of varying hydrographic conditions that affect irradiation, pH as well as concentrations of dissolved oxygen, sulfate, hydrogen sulfide, nutrients and metals availability [52–54, 57]. In this discussion, we first consider the challenges associated with the analytical techniques for detection of zero-valent sulfur used in this work. We then review the seasonal changes in the lake chemical composition, with an emphasis on sulfur cycle and its effects on the isotopic composition of sulfur species. Finally, we discuss the relevance of Lake Kinneret as an analog for the Proterozoic Ocean.

### Lake mixis

Temperature and conductivity profiles are good indicators of the stratification of the water column. The annual conductivity changes in Lake Kinneret water column are caused by a combination of thermal stratification, evaporation, and the inflow of both fresh and saline water. Fresh water onshore sources (i.e. Jordan River, runoff, rain etc.) contribute approximately 89% of the total inflow while the saline springs at the bottom of the lake contribute to the remaining 11% [1]. The less dense fresh water tends to remain in the epilimnion, thus decreasing its conductivity, while the saline water increases the conductivity of the hypolimnion [85]. In March (LK1), the conductivity was constant through the entire water column, implying that the water column was completely mixed (Figure 2a). During the mixed period (LK1, Figure 2b), the pH at the surface was slightly higher than at the bottom (0.41 pH units) due to photosynthesis in the photic layer, which decreases carbon dioxide concentrations. Indeed, pe values are consistent with a carbonate-buffered system in equilibrium with the atmosphere [53]. The low iodide concentrations (<11 nM) measured during the mixis period are consistent with the oxidation of iodide, produced by microbial mineralization of organic matter, to iodate by dissolved oxygen [34] or by microbial oxidation of iodide to iodate by nitrifying bacteria [32, 33]. During this time period, the water column is oxic and microbial sulfate reduction in the water column does not occur. Hydrogen sulfide, which is produced by microbial sulfate reduction in the chemocline of Lake Kinneret, is not detectable and is likely only produced within the sediments. During the LK1 sampling, no sulfide oxidation intermediates were detected, even in the bottom waters (Figures 4g, 5a). This indicates that sulfide oxidation intermediates that may be produced in the sediments are further oxidized to sulfate fast enough to prevent their diffusion to the overlying waters of Lake Kinneret. Indeed, sulfate accounted for >99.9% of total analyzed sulfur species in the lake during mixis (Table 2).

**Table 2:** Depth integrated inventory of sulfur

Sampling	H_2_S (mol S m^−2^)	SO_4_^2−^ (mol S m^−2^)	SO_3_^2−^ (mol S m^−2^)	S_2_O_3_^2−^ (mol S m^−2^)	S^0^(HCN) (mol S m^−2^)	S^0^(CHCl_3_) (mol S m^−2^)	Sum (mol S m^−2^)
LK1	0 (<0.01)	20.7 (100.0)	0 (<0.01)	6.48 × 10^−5^ (<0.01)	0 (<0.01)	0	20.7
LK2	1.71 × 10^−2^ (0.09)	19.8 (99.9)	7.32 × 10^−3^ (0.04)	1.01 × 10^−3^ (<0.01)	1.82 × 10^−3^ (0.01)	3.27 × 10^−4^	19.8
LK3	1.86 (8.99)	18.8 (90.8)	7.90 × 10^−3^ (0.04)	2.79 × 10^−2^ (0.13)	2.13 × 10^−3^ (0.01)	1.01 × 10^−3^	20.7
LK4	3.40 (16.4)	17.3 (83.3)	1.03 × 10^−2^ (0.05)	1.40 × 10^−2^ (0.07)	3.24 × 10^−2^ (0.16)	1.85 × 10^−3^	20.8
LK5	3.65 (18.2)	16.3 (81.5)	5.27 × 10^−3^ (0.03)	2.70 × 10^−3^ (0.01)	4.66 × 10^−2^ (0.23)	6.41 × 10^−3^	20.0
LK6	9.37 × 10^−2^ (0.45)	20.7 (99.1)	4.03 × 10^−3^ (0.02)	2.85 × 10^−2^ (0.14)	6.56 × 10^−2^ (0.31)	7.65 × 10^−2^	20.9

Numbers in parentheses stand for a percent of total analyzed sulfur. Thiosulfate concentrations were multiplied by a factor of two in order to calculate sulfur concentrations. Only cyanide-reactive zero-valent sulfur (and not chloroform-extractable sulfur) was taken into account in calculation of the sum of sulfur species concentration and total sulfur pool. The relative variation in the sum of the sulfur species is below 0.4%.

In our calculations we defined sulfide (product) as A, and sulfate (reactant) as B.

### Early stage of stratification

Lake Kinneret thermal stratification usually begins in April. In our campaign the thermal stratification was first detected in the May (LK2) sampling (Figure 2c). Following the onset of stratification (LK2 sampling, Figure 4b), hypolimnetic dissolved oxygen and nitrate were depleted due to heterotrophic microbial activity [57] and sulfate reduction took over, leading to the gradual accumulation of hydrogen sulfide during the stratified period [54]. The pH was elevated in the epilimnion (Figure 2d) due to photosynthesis and a clearly visible redoxcline formed due to depletion of oxidized nitrogen compounds, followed by further decrease in pe due to formation of sulfide below a depth of 33.1 m. LK2 sampling was the only one in which the thermocline did not coincide with the chemocline (Figure 2c). The thermocline was not sharp while the chemocline indicated the upper border of the benthic boundary layer [86]. In the oxic part of the epilimnion, iodide was depleted to concentrations were below the MDL (Figure 3b). In anoxic non-sulfidic waters, iodide concentration slowly increased with depth, while in hydrogen sulfide-rich bottom layer iodide concentrations increased sharply. This profile is typical for stratified systems characterized by diffusion of iodide from sediments, where it is produced by decomposition of organic matter, and its consumption in the oxygen-rich epilimnion [34]. During that period the hydrogen sulfide was detected only in the bottom layer of the lake. The highest detected concentration of hydrogen sulfide in the water column was only 8.0 µM and sulfate accounted for 99.9% of total analyzed sulfur (Table 2).

### Shallow stable stratification

The depth of the thermocline decreased from spring to mid-summer. During our July sampling period (LK3), the thermocline was situated in the photic zone. These conditions are favorable for the bloom of phototrophic sulfur bacteria that oxidize hydrogen sulfide anaerobically to sulfur and sulfate [55]. The concentration of bacterial chlorophyll in this season at the chemocline was below detection limit (not shown). This was a rather unexpected observation as a bloom of brown sulfur bacterium *Chlorobium phaeobacteroides* is usually observed during this season and the chemocline was situated in the photic water layer [55]. A sharp increase in iodide concentrations above the chemocline coincided with sharp decrease in dissolved oxygen concentration (Figure 4c), indicating the flux of iodide from sediments into the relatively well-mixed anoxic hypolimnion. In July (LK3 sampling), dissolved oxygen was detectable until a depth of 11 m while the sulfide penetration depth was 11.5 m (Figure 4c). In this sampling zero-valent sulfur concentrations (Figure 4i) were lower than the concentrations of thiosulfate and sulfite (Figure 5c). Sulfate accounted for 90.8%, sulfide for 9.0%, and S_n_O_3_^2−^ sulfur (sum of thiosulfate and sulfite sulfur) for 0.17% of total analyzed sulfur (Table 2). We interpret these results as demonstrating the relative instability of the anomalously shallow chemocline. Thus relatively high concentrations of sulfur oxyanions may be explained by a recent mixing event between oxygen and sulfide rich layers. Low concentration of zero-valent sulfur may be explained as well by the absence of phototrophic sulfide oxidizing bacteria bloom during that time period.

### Deep stable stratification

In the summer months, the thermocline deepened under the influence of the rising epilimnion water temperature and increasing wind shear. During our October sampling (LK4), the thermocline was still stable, but it was situated deeper than in the summer (Figure 2g). Epilimnetic pH and pe were high due to photosynthesis, whereas hypolimnetic pH was low due to anoxic respiration and pe was low due to the buildup of sulfide. The chemocline was detected at 18.5 m depth. This period was characterized by the continuous accumulation of hypolimnetic sulfide as the result of microbial sulfate reduction in the water column and in the sediment that is supported by the influx of organic material sinking from the photic zone [54].

The profiles of sulfate and sulfide in October differed from their shape at other seasons. The local maximum in hydrogen sulfide concentration and local minimum in sulfate concentrations were detected 1 m below the chemocline (Figure 4d). We explain these features by the fast microbial sulfate reduction of sulfate below the chemocline. This explanation is supported by the unusual shape of the iodide profile. The highest concentration of iodide was detected 1 m below the chemocline, coinciding with the local minimum in sulfate concentrations (Figure 3d), and not in the bottom layer of the lake. High concentrations of iodide point to fast mineralization of organic matter at this redox transition within the water column. Additional processes that may promote the iodide maximum include the reduction of iodate in the sulfidic-oxygenated water interface by sulfite, thiosulfate and sulfide [36, 37]. This assumption is supported by the high concentrations of sulfite, thiosulfate, and sulfide found at the chemocline.

During the October sampling, zero-valent sulfur concentrations (Figure 4j) were higher than thiosulfate and sulfite (Figure 5d). Sulfate accounted for 83.3%, sulfide for 16.4%, S_n_O_3_^2−^ for 0.12% of the total analyzed sulfur, and S^0^ for 0.16% of the total sulfur (Table 2). The ratio of zero-valent sulfur to thiosulfate concentrations was higher than in the previous sampling periods, either as a result of intense phototrophic microbial formation of elemental sulfur, given that the chemocline was located in the lower boundary of the photic zone, or due to the high hydrogen sulfide formation rates below the chemocline [12]. The latter may have led to an increase in hydrogen sulfide to dissolved oxygen ratios.

### Early stage of lake mixing

With the decline in air temperatures in autumn, surface water temperatures decrease and the conductive mixing increases, leading to the deepening of the thermocline until Lake Kinneret water column finally mixes around December-January [1]. Through the late autumn, the thermocline deepens step-wise during storm events. A criterion of a mean thermocline gradient >0.3°C m^−1^ between the depths of 10 and 35 m was defined to determine the stability of the water column [87]. While the stabilization of the water column may be in some cases fortified by the salinity gradient, according to this criterion, the water column stratification was metastable during the December (LK5) sampling (Figure 2i). The gradient decrease in pH in the thermocline was less pronounced in the December sampling than in October (Figure 2j) possibly due to a lower primary production in the epilimnion after the end of the algal blooms. Although pe profiles were similar during both periods, the local maximum of iodide concentrations below the chemiocline was much less pronounced than in the previous sampling and was not accompanied by either maximum in hydrogen sulfide concentrations or minimum in sulfate concentrations. The absence of the iodide peak is probably due to the deepening of the chemocline. Increase in iodide concentrations in the surface waters may be attributed either to photolytic decomposition of organic matter or to microbial reduction of iodate. Sulfate accounted for 81.5%, sulfide for 18.2%, S_n_O_3_^2−^ for 0.04%, and S^0^ for 0.23% of the total analyzed sulfur (Table 2). Although this sampling was performed at the beginning of the new mixis period, the inventory of sulfide at point A represented the annual maximum (3.65 mol S m^−2^) detected in the lake (Table 2).

### Advanced stage of lake mixing

In early winter, the chemocline deepened following the erosion of the thermocline (LK6, Figures 2k, 4f). In January, the water column was on the verge of mixing, as the temperature difference between epilimnion and hypolimnion was only 0.34°C, but a very sharp conductivity gradient deep in the water column (approximately 2.0 m above bottom) marked a border between the epilimnion and hypolimnion (Figure 2k). The pH decrease at the thermocline was much less pronounced than in the previous sampling periods, but the pe still decreased sharply at a depth of 38 m due to the high concentration of hydrogen sulfide in the bottom waters (Figure 2l). Simultaneously, iodide concentrations decreased in the epilimnion as well as in the hypolimnion, likely due to the oxidation of iodide by dissolved oxygen during the active mixing phase of the water column. Around the chemocline, dissolved oxygen co-existed with hydrogen sulfide (Figure 4f), and the mixing of oxic and sulfidic waters led to formation of relatively high concentrations of zero-valent sulfur (Figure 4l), thiosulfate, and sulfite (Figure 5f). Sulfate accounted for 99.1%, sulfide for 0.45%, S_n_O_3_^2−^ for 0.16%, and S^0^ for 0.31% of the total sulfur (Table 2). During this period, the inventory of sulfide oxidation intermediates ([S^0^] + [SO_3_^2−^] + 2 × [S_2_O_3_^2−^]), 0.098 mol S m^−2^, was slightly higher than the inventory of sulfide, 0.094 mol S m^−2^ (Table 2).

### Isotope composition of sulfur species

**Figure 7:**
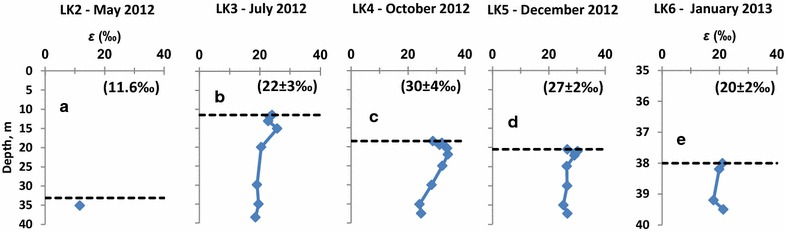
Seasonal variations in the depth profiles of the isotopic fractionation between sulfate and sulfide (*ε*) in Lake Kinneret.* Black dashed lines* denote the depth of the chemocline.** a** LK2 sampling,** b** LK3 sampling,** c** LK4 sampling,** d** LK5 sampling,** e** LK6 sampling. Please notice difference in depth scale on panel** e**. Numbers in parentheses represent the average isotope fractionation between sulfate and sulfide and its standard deviation.

In May, at the beginning of the stratification period, the sulfur isotope fractionation between sulfate and sulfide was *ε* = 11.6 ‰ (Figure 7a). During the LK3, LK4, and LK5 samplings, which are characterized by stable chemocline conditions, the depth profiles of ε have similar shapes (Figure 7b–d). In these profiles the highest *ε* values, 25.8, 34.0, and 30.2 ‰ were detected at 3.5, 3.5, and 0.5 m depths, below the chemocline. *ε* values at the chemocline were 1.6–5.2 ‰ lower than the higher *ε* values deeper within the water column. The average values of *ε* from all horizons of the same sampling differ throughout the year (Figure 7, numbers in parentheses). The highest average ε (30 ± 4 ‰) was detected in the LK4 sampling performed in October. During earlier seasons and during the de-stratification of the lake, lower sulfur isotope fractionations were measured.

The shape of the *ε* profile, characterized by increase of *ε* with depth below the chemocline followed by decrease toward the bottom of the lake may be explained in two ways. The first explanation is based on the lower sulfate concentration in the bottom waters, which may result in lower isotope fractionation by sulfate reducing bacteria [51, 68]. Alternately, microbial sulfate reduction may be accompanied by microbial or chemical disproportionation of sulfur and thiosulfate produced by chemical and microbially assisted oxidation of hydrogen sulfide. Disproportionation processes are known to increase isotope fractionation between sulfate and sulfide [40]. Two factors allow microbial disproportionation of sulfide oxidation intermediates below the chemocline.

The first factor is an increase in bioavailability of zero-valent sulfur due to the reaction of elemental sulfur with hydrogen sulfide, which results in the formation of water soluble inorganic polysulfides (Eq. 9).

9 $$ {\text{HS}}^{-} + \left({{\text{n}} - 1} \right)/ 8 {\text{ S}}_{ 8} = {\text{H}}^{+} + {\text{S}}_{\text{n}}^{ 2-} $$

Although polysulfides were not detected in the water column, analyses performed by a more sensitive, although less robust method [88], suggested that polysulfides are present in Lake Kinneret water column at the hundredth of pM concentrations [59]. The second factor is the relatively high concentration of thiosulfate measured: up to 0.18–5.54 µM, depending on the season.

In October (LK4), the lake stratification was stable and high rates of bacterial sulfate reduction in the chemocline resulted in local minimum of sulfate concentration and local maximum of sulfide concentration. These processes left their footprint on the sulfur isotope composition (Figure 6c). A sharp increase in the δ^34^S_SO4_, followed by decrease in δ^34^S_SO4_ with depth was likely due to higher fraction of sulfate being reduced in the chemocline. In December (LK5), a similar profile of ε as a function of depth was recorded (Figure 7e). The highest value of ε = 30 ‰ was detected at 21 m depth, while ε at the chemocline and near the bottom is 25–27 ‰. During this sampling period, chloroform-extractable zero-valent sulfur in the chemocline was heavier than sulfide by 3.1 ‰. This effect may be explained either by bacterial oxidation of sulfide to elemental sulfur [89 and reference therein] or by partial equilibration between hydrogen sulfide and elemental sulfur due to formation of inorganic polysulfides (Eq. 9) [90]. Kinetic studies of this reaction (Eq. 9) under controlled conditions and in natural aquatic systems show that equilibrium is reached in seconds in the hydrogen sulfide-polysulfide-dissolved sulfur system [91], in minutes to hours in the hydrogen sulfide-polysulfide-colloidal sulfur system [92, 93], and in hours or longer in the hydrogen sulfide-polysulfide-rhombic sulfur system [94]. A scenario, which combines these two processes, includes the formation of elemental sulfur with unknown isotopic composition by microbial oxidation of hydrogen sulfide, followed by a shift in the isotopic composition of elemental sulfur to δ^34^S values slightly larger than those of sulfide by (at least partial) equilibration with polysulfides (Eq. 9).

During disruption of the stratification (LK6, January 17), the isotopic fractionation decreased to *ε* = 18–21 ‰. These values do not necessarily support presence of microbial sulfur disproportionation in the lake. Although the highest concentrations of sulfide oxidation intermediates (up to 53 µM zero-valent sulfur and up to 5.5 µM thiosulfate) were detected, the sulfur isotope fractionation was relatively low, possibly, due to the formation of relatively isotopically light sulfate due to massive reoxidation of isotopically light hydrogen sulfide. During this sampling period, zero-valent sulfur in the hypolimnion was 1.1–3.3 ‰ heavier than sulfide. At the chemocline the difference between δ^34^S of zero-valent sulfur and sulfide was as low as 0.6 ‰. We interpret these values as partial equilibration between zero-valent sulfur and sulfide through polysulfide formation in the hypolimnion and lack of such equilibration at the unstable chemocline during the period of mixing of the lake water layers.

**Figure 8:**
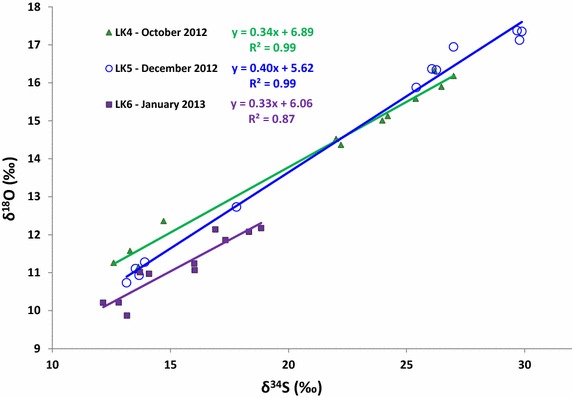
Seasonal variations in the sulfur vs. oxygen isotope composition of sulfate in Lake Kinneret. Formula represent equations of the corresponding linear regressions. See “Discussion” on the observed trends in the text.

Relative variations in the δ^34^S_SO4_ and δ^18^O_SO4_ may provide further insights into the pathway of microbial sulfate reduction in Lake Kinneret. The slopes of the δ^34^S_SO4_ vs. δ^18^O_SO4_ plot (Figure 8) calculated for the LK4 (0.34 ± 0.01), LK5 (0.40 ± 0.01), and LK6 (0.33 ± 0.04) sampling periods are moderate relative to steeper slopes that can be found in marine sediments [83]. We excluded LK3 sampling from this discussion as it was performed soon after stratification and only a minor fraction of sulfate was reduced, resulting in a high variability in slope (RSD = 39%) that was not reliable. The moderate slopes measured at LK4, LK5, and LK6 suggest that the isotopic composition of both oxygen and sulfur in sulfate varies due to large sulfate uptake by microbial cells, and usually characterize environments with high sulfate reduction rate (higher than 1 μmol cm^−3^ year^−1^). Slopes of this range have previously been found in estuaries and cold seeps [95, 96]. Calculating sulfate reduction rates in Lake Kinneret is exceptionally difficult, due to mixing by high eddy diffusion in the lake, sulfate diffusion in and out of the sediment, and redox reactions involving sulfide oxidation or sulfur disproportionation that may mitigate any change in sulfate concentration. Due to the low concentration of sulfate in the lake (less than 600 μM), it is possible that sulfate supply limits the rate of microbial sulfate reduction. Similar to a previous study [83], we suggest that the low slopes in the cross plot of δ^34^S_SO4_ vs. δ^18^O_SO4_ in Lake Kinneret indicate a low intracellular rate of reoxidation of sulfide oxidation intermediates back to sulfate, as is common during high rates of sulfate reduction.

Finally, during the LK6 sampling, when active oxygenation of sulfidic waters took place, the δ^18^O_SO4_ was shifted to lower values compared to LK4 and LK5. This may be due to the intensification of sulfide oxidation explained by the sinking of the chemocline, decrease in the concentration of sulfide, and simultaneous increase in the concentration of sulfate in the deep waters (Figure 4e, f). During sulfide oxidation the oxygen atoms in the resulting sulfate derive from water if the oxidation is purely anoxic, and partially from water and partially from atmospheric oxygen if the oxidation happens near the surface. Given the δ^18^O of the lake waters, the lower δ^18^O_SO4_ values could result from a combination of anoxic and oxic sulfide oxidation.

### Lake Kinneret as an analog of Proterozoic ocean

Sulfate concentrations, as far as they are able to be reconstructed, have undergone significant variations throughout Earth history. Our knowledge of sulfate concentrations is based on three approaches: measuring the magnitude and the rate of change of the sulfur isotopic composition of sedimentary sulfur species such as pyrite, carbonate associated sulfate (CAS), and sulfate evaporite minerals, measuring the concentration of sulfate in fluid inclusions in evaporite minerals [97], and the more qualitative approach of assessing the presence and extent of sulfate evaporites [98]. Sulfur isotope fractionation during bacterial sulfate reduction is ranging between −3 and 66 ‰ [68, 99, 100]. At sub-millimolar sulfate concentrations, the absolute value of fractionation decreases as a function of sulfate concentration [51]; this isotopically light sulfur can then be incorporated into sedimentary pyrite and may be utilized to estimate seawater sulfate concentrations in the ancient ocean. Similarly, the sulfur isotope composition of marine sulfate, which reflects the balance of the sources and sinks of sulfate to the ocean, can change as a function of changes in the sulfur biogeochemical cycle. Fast rates of change in the sulfur isotope composition of sulfate have been used to argue that sulfate concentrations must have been correspondingly low [101]. Sulfate concentrations can also be directly measured in fluid inclusions, but depend on an assumed concentration of calcium and therefore are less reliable [97]. Finally, the precipitation of gypsum before halite in ancient shallow-marine evaporite deposits requires ≥2.5 mM sulfate concentration and thus may be also used for a rough evaluation of sulfate concentrations [98].

Despite these rather poor proxies for sulfate concentration over Earth history, our basic understanding is that marine sulfate concentrations follow the concentration of atmospheric oxygen, partially because oxidative weathering of pyrite is one of the main sources of sulfate to the ocean [102]. Based on the low absolute isotope fractionation between sulfide and sulfate in sedimentary rocks (<15 ‰), sulfur isotope systematics of volcanogenic massive sulfide ore deposits [65], and an absence of significant sulfate evaporites [98 and references therein], marine sulfate concentrations of 80–200 µM are suggested to have existed in the Archean ocean before 2.5 billion years ago [51, 65]. In turn, the ocean water column during the Archean has been suggested to be ferruginous, e.g. to contain abundant dissolved iron (II) and no dissolved oxygen or hydrogen sulfide [103].

Following the rise of atmospheric oxygen concentrations c.a. 2.4 Gyr, sulfate concentrations in the ocean would have risen to ≥2.5 mM by c.a. 2.22–2.06 Gyr (Lomagundi event) due to the initial oxidation of sulfur contained in surface rocks [98, 104]. As the ocean redox state adjusted to sustained, if potentially low, oxygen production after the Lomagundi event, sulfate concentrations in the ocean likely dropped, possibly to sub-millimolar level, as estimated from the isotopic composition of coexisting pyrite and sulfate [49, 104, 105]. Sulfate concentrations of 0.5–4.5 mM have been suggested for the Mesoproterozoic ocean [101]. It is thought that hydrogen sulfide was abundant in the deep Proterozoic ocean [66] or at least in mid-depth near-shore waters [105]. During the Neoproterozoic (800–550 million years ago), sulfate concentrations in the ocean were likely higher than during the Mesoproterozoic, perhaps between 0.8–10.1 mM [101]. Sulfur isotope fractionation between sulfate and sulfide increased at this stage from <15 ‰ to >15 ‰ in the early Paleoproterozoic [106] and further increased to larger than 45 ‰ in the Neoproterozoic [107]. The latter increase is attributed to the disproportionation of sulfide oxidation intermediates, which started to form in larger quantities due to an increase in oxidation state of the planet [107, 108]. The next increase in sulfate concentrations is associated with an increase in oxygen concentrations that occurred during the latest Neoproterozoic. At this stage (the early Ediacaran) the sulfur isotope fractionation between sulfate and sulfide reached ≥65 ‰ [109].

Lake Kinneret represents a unique natural laboratory for studying the sulfur cycle in a stratified aquatic system with sub-millimolar concentrations of sulfate. The concentration of aqueous sulfate in the lake during the winter mixis period (c.a. 550 µM) and the presence of free hydrogen sulfide in the hypolimnion during the stratification period correlate well with our understanding of global ocean chemistry with respect to the sulfur species in the late Paleoproterozoic and Mesoproterozoic. The evolution from the ferruginous Archean ocean to the sulfidic deep-water Proterozoic ocean occurred most likely at sulfate concentrations similar to those existing today in Lake Kinneret. In order to estimate the impact of iron on the sulfur cycle in the water column of the lake, we compared the sulfide and iron budgets. Total iron concentrations in the lake are always lower than 1.2 µM [84], and iron sedimentation rates are estimated to be 200–500 ton/year which corresponds to <9 × 10^6^ mol/year. The average concentration of sulfide in the hypolimnion (200 µM) may be estimated from the data presented in Table 2, and the width of the anoxic layer in summer and autumn (at least 18 m). Together with the volume of hypolimnion of Lake Kinneret during the stable stratification period (10^9^ m^3^ [110]), a minimum hydrogen sulfide production rate of 2 × 10^8^ mol/year can be estimated from these data. As part of the sulfide is re-oxidized to intermediate valence state sulfur species and sulfate during the stratification period, the actual rate of sulfide formation should be even higher. Thus, a ratio between the minimum sulfide production rate and the maximum iron sedimentation rate in Lake Kinneret is estimated to be 23 mol/mol. An excess of sulfide production over iron sedimentation in the lake implies that precipitation of iron sulfides in the water column should have a minor effect on both the hydrogen sulfide budget and its isotopic composition in the water column.

The combination of sub-millimolar sulfate concentrations with relatively high concentrations of sulfide oxidation intermediates that are prone to microbial disproportionation (e.g. zero-valent sulfur and thiosulfate, Figures 4, 5) should result in relatively high sulfur isotope fractionation in Lake Kinneret (up to 34 ‰), higher than commonly measured in Paleoproterozoic sedimentary rocks, yet lower than the highest values from the Neoproterozoic sedimentary record. This apparent discrepancy between the sulfur isotope fractionation observed in Paleoproterozoic sedimentary rocks versus that in Lake Kinneret may be explained as follows.

First, the difference in hydrographic settings between the global Proterozoic ocean and Lake Kinneret may generate different sulfur isotope fractionation between sulfate and hydrogen sulfide as a result of variations in chemical and biological processes at the redox interfaces. The availability of light represents one of the factors which may affect sulfur isotope fractionation. Production of significant amounts of elemental sulfur by phototrophic oxidation of hydrogen sulfide, may, for example, fuel sulfur disproportionation (Eq. 6) and increase the sulfur isotope fractionation between sulfate and hydrogen sulfide [40]. The water column of Lake Kinneret is relatively turbid however, and the photic layer extends only to c.a. 15 m depth [111]. Thus, the chemocline was situated in the photic zone only during one of sampling periods, in July (LK3). Indeed, our results show that the concentration of zero-valent sulfur (Figure 4) and the isotope fractionation between sulfate and sulfide (Figure 7) are not elevated in the chemocline during this sampling, suggesting that disproportionation is not significant in this system. The higher concentration of dissolved oxygen in the epilimnion of Lake Kinneret compared to the surface waters of the Proterozoic ocean represents another difference which may result in formation of relatively high concentrations of sulfide oxidation intermediates. During autumn and early winter, a combination of high concentrations of dissolved oxygen in the epilimnion and of hydrogen sulfide in the hypolimnion results in a high inventory of elemental sulfur in the chemocline and hypolimnion (Figure 3). The role of microbial disproportionation of intermediate sulfur species in the generation of relatively high isotope fractionation between sulfate and hydrogen sulfide was not resolved unequivocally by this study. Measurements of triple sulfur isotope composition (e.g. δ^33^S and δ^34^S) of sulfate, hydrogen sulfide and zero-valent sulfur may clarify the relative contribution of microbial sulfate reduction and sulfur disproportionation to sulfur isotope fractionation in the Lake Kinneret water column. Application of such analysis may as well reveal if in natural aquatic systems with sub-millimolar sulfate concentrations sulfur isotope fractionation between sulfate and sulfide of >30 ‰ may be produced by microbial sulfate reduction alone.

Second, translation of the sulfur isotopic signal from the water column to the sediment may affect the isotopic composition of pyrite preserved in the sedimentary record [63]. We can envision two scenarios. In the first scenario, significant amounts of hydrogen sulfide are produced in sedimentary pore-waters that are depleted in sulfate relatively to the water column, and no significant precipitation of iron sulfide or pyrite occurs in the water column. Hydrogen sulfide diffuses from the sediment into the water column, and sulfate diffuses from the water column into the sediment. In this case hydrogen sulfide in the pore-waters may be enriched with ^34^S, as it is produced from sulfate, which is itself enriched with ^34^S due to Rayleigh distillation as well as the low sulfate-sulfide sulfur isotope fractionation at low sulfate concentration. As this ^34^S-enriched hydrogen sulfide is preserved in the sediment, the sulfur isotope fractionation between sulfate and sulfide should be lower than in the water column. In turn, hydrogen sulfide produced in the water column should be mixed with hydrogen sulfide which diffuses from the sediment. This sulfide pool should be eventually completely reoxidized to sulfate, such that its isotopic composition should not be preserved in the sediment. In the second scenario, hydrogen sulfide as well as sulfate diffuses from the water column into the sediment. Therefore, a mixed isotopic signal of ^34^S-enriched sulfide produced in the sediment and relatively light water column sulfide should be preserved in the sediment. This scenario is not relevant for Lake Kinneret, however, as hydrogen sulfide concentrations increase with depth in its uppermost sediment layer [112]. In both scenarios, sedimentary pyrite sulfur is to be expected to be isotopically heavier than water column hydrogen sulfide. Measurement of the sulfur isotope fractionation between sulfate and sedimentary pyrite is a promising future direction of this research.

## Conclusions

Monomictic Lake Kinneret with sub-millimolar concentrations of sulfate in the water column was thermally stratified from May 2012 to January 2013. At the deepest point of the lake, the sulfate inventory decreased by 21% between March and December due to microbial sulfate reduction (Table 2). Simultaneously, the depth of the chemocline decreased between May and July and increased between July and January (Table 1). As a result, hydrogen sulfide concentrations in the hypolimnion reached in December about 45% of the sulfate concentration measured during the mixis of the lake (Figure 4) and then decreased back to <10% of the epilimnetic sulfate concentration on January 17. Simultaneously, iron concentrations were rather low in the water column. These findings suggest that significant oxidation of sulfide is ongoing at the chemocline. Of the different sulfide oxidation intermediates, the presence of thiosulfate, sulfite, and zero-valent sulfur was documented in the water column of the lake, while concentrations of inorganic polysulfides, tetrathionate, and thiocyanate were below the detection limit. During the early stage of the stratification (May and July), sulfur oxyanions (e.g. thiosulfate and sulfite) were the main products of the incomplete oxidation of hydrogen sulfide. During the stable stratification and mixing periods, however, zero-valent sulfur prevailed over sulfur oxyanions (Table 2). These findings indicate that the lack of mixing between the epilimnion and hypolimnion and the relatively small concentrations of hydrogen sulfide in the spring limited the buildup of sulfide oxidation intermediates. In autumn, the mixing of hydrogen sulfide and dissolved oxygen increase and the concentration of hydrogen sulfide was much higher which led to the formation of more reduced sulfide oxidation intermediates (e.g. ZVS). On the other hand we cannot rule out that changing bacterial community affected consumption rates of various sulfur species leading to a shift in sulfur speciation [8]. During the terminal stage of lake water column mixing (January), the water column inventory in sulfide oxidation intermediates was even higher than the hydrogen sulfide inventory.

The sulfur isotope fractionation between zero-valent sulfur and hydrogen sulfide ranged between 0.9 and 3.2 ‰. This effect may be explained by bacterial oxidation of sulfide to elemental sulfur, by partial equilibration between hydrogen sulfide and elemental sulfur due to formation of inorganic polysulfides, or by a combination of these processes. The sulfur isotope fractionation between sulfate and hydrogen sulfide in the water column increased from the lowest value of 11.6 ‰ in May to 30 ± 4 ‰ in October and then decreased to 20 ± 2 ‰ in January. The highest fractionation observed is at the upper limit of fractionation by microbial cultures at corresponding sulfate concentrations. The presence of sulfide oxidation intermediates in the water column suggests microbial disproportionation of sulfide oxidation intermediates was ongoing, but the existence of this process in the water column could not be unequivocally confirmed by our data. Seasonal variations in hydrographic, chemical, and biological processes in Lake Kinneret provide a unique opportunity to study the cycling of sulfur in a system with sulfate concentrations similar to those suggested to have existed in the Proterozoic Ocean. Results of this research suggest that other factors besides low sulfate concentrations alone are required for low sulfur isotope fractionation between sulfate and pyrite in ancient sedimentary record. Such factors may include an absence of quantitatively important microbial disproportionation of sulfide oxidation intermediates as well as a change in the isotopic composition of sulfur species formed in the water column during its transport to the sediment.

## Methods

### Sampling

The sampling was carried out at station A., situated in the central part of Lake Kinneret at a maximum water depth of c.a. 42 m (Figure 1). The physicochemical conditions in the water column were characterized by an in situ multi-probe (see below). Results of these in situ measurements were used to identify the chemocline and the target depths for our water column profiles (Table 1). The latter was planned to maximize resolution in the vicinity of the chemocline in addition to sampling the hypolimnion and epilimnion. Samples from metalimnion and hypolimnion were pumped from the target depth using a peristaltic pump (Masterflex E/S Portable Sampler, Cole-Parmer) fitted with a 3/8″ PVC tube (Sigma-Aldrich). The tube inlet was attached to the in situ multi-probe to simultaneously obtain accurate measurement of the sampling depth and general system parameters. The well mixed epilimnion was sampled by a 5 L Niskin bottle. The samples were preserved immediately onboard of the ship as described below to minimize their re-oxidation by atmospheric oxygen.

### Quantitative analysis

Depth, temperature, and dissolved oxygen, pH, pe, [H_2_S] were measured by a modified version of the in situ multi-probe introduced by Eckert et al. [56] with on-line data processing. This probe is equipped with pe, pH, and pH_2_S electrodes (Ingold, Taunusstein F.R.G), dissolved oxygen, temperature (Orbisphere Laboratories, Switzerland) and depth sensor. The multi-probe dissolved oxygen and pH_2_S measurements were used for the sole purpose of locating the depth of the chemocline at the initial stage of the sampling. Data obtained by the multi-probe is not presented. Conductivity was measured on board in water samples on a Wissenschaftlich-Technische Werkstätten GmbH (WTW), LF 91 conductivity meter. Dissolved oxygen was measured by the Winkler assay in discrete samples [113, 114]. Dissolved iron concentrations were determined using the ferrozine method [115]. Ferrozine was added to the samples in the field, and samples were kept chilled in the dark until dissolved iron concentrations were measured by spectrophotometry in the laboratory. The MDL of the method is 1 µM.

**Figure 9:**
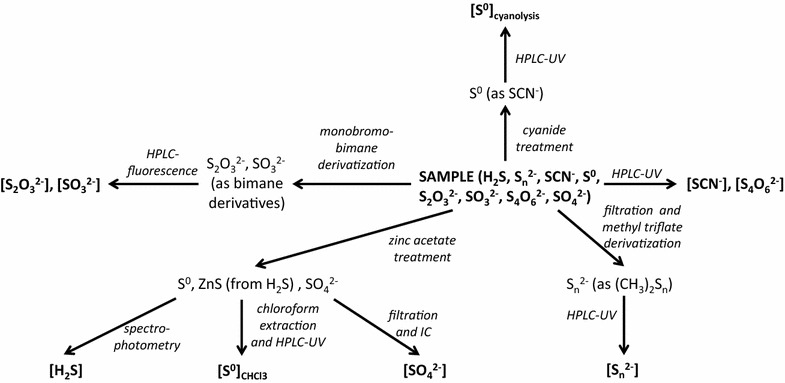
Sample processing scheme for the analysis of the concentration of sulfur species.

The sample processing scheme for the analysis of the concentrations of sulfur species is presented in Figure 9.

Total S(II) concentrations were measured by spectrophotometry in unfiltered samples (Figure 9) [116]. The samples were preserved in the field by the addition of zinc acetate 200 gL^−1^ solution in a ratio of 1:50 (Figure 9). The MDL of this method is 1 µM. Total S(II) analyzed by this method includes protonated and deprotonated forms of free sulfide, acid-reactive particulate metal sulfides (e.g. iron and manganese sulfides) as well as S(II) moiety of protonated and deprotonated polysulfide species.

For all the chromatographic methods described in the following section, the MDL signal to noise ratio (S/N) is 3. Iodide concentrations in unfiltered samples were determined by HPLC (1260 Infinity, Agilent Technologies) using a polyethylene glycol modified reversed-phase C_30_ column (Nomura Chemical, Develosil 5 µ RPAQUEOUS, 150 × 4.6 mm I.D) and an eluent composed of 300 mM sodium sulfate and 50 mM sodium chloride. Flow rate was 1.0 mL min^−1^ and UV detection at a wavelength of 220 nm was used to quantify iodide [117]. The MDL of this method is 7 nM. Sulfate concentrations were measured by ion chromatography (IC; Dionex, DX500) in the supernatant of samples pretreated in the field with zinc acetate (200 gL^−1^ solution in a ratio of 1:50) to precipitate any sulfide present (Figure 9). A guard column (AG4A-SC), anion exchange column (AS4A-SC) and suppressor (ASRS-300) were used for these analyses. The mobile phase was composed of 1.8 mM sodium carbonate and 1.7 mM sodium bicarbonate mixture, and the flow rate was 2.0 mL min^−1^. Duplicate analyses were conducted and relative standard deviation (RSD) on peak area of 0.14% was calculated. The MDL of the method is 10 µM. Inorganic polysulfides in samples filtered through 0.2 μm pore-sized filters (Figure 9) were converted to respective dimethylpolysulfanes by rapid single-phase derivatization with methyl trifluoromethanesulfonate in the field followed by preconcentration by extraction with n-dodecane in the laboratory. Then concentrations were determined in triplicate analyses by HPLC. The eluent was composed of 90% methanol and 10% water at flow rate 1 mL min^−1^. A C_18_ reversed-phase column (Grace, Prevail C18 5 μm, 250 × 4.6 mm I.D) with UV detection at wavelengths of 220 and 230 nm was used to quantify dimethylpolysulfanes [118]. The MDL of this method is 0.1–0.4 µM for individual polysulfide species depending on the chain length. Concentrations of polysulfides were below MDL in all samples. Concentrations of chloroform extractable zero-valent elemental sulfur were determined by the chloroform extraction method [119] with minor changes. The samples were preserved in the field by the addition of zinc acetate 200 gL^−1^ solution at a ratio of 1:50 (Figure 9), and extracted in the laboratory by triple extraction with chloroform (2 mL and 2 × 1 mL). The chloroform-extractable sulfur was analyzed by HPLC using a C_18_ reversed-phase column (Grace, Prevail C18 5 μm, 250 × 4.6 mm I.D). The eluent was composed of 100% methanol with a flow rate of 1 mL min^−1^ and UV detection at 220 and 230 nm wavelengths was used to quantify elemental sulfur. Triplicate analyses were conducted; the MDL of this method (including preconcentration by extraction) is 80 nM. Cyanide-reactive zero-valent sulfur concentrations were determined in unfiltered samples (Figure 9) using an improved cyanolysis protocol carried out in the field [46]. The concentration of thiocyanate in the samples was later determined in the laboratory by the same method as was used for iodide quantification (see above). The MDL of this method was c.a. 30 nM, depending on the final volume of pre-concentrated sample. Naturally occurring thiocyanate concentrations were determined using the same method without pretreatment in the field. RSD = 3%, MDL of the method is 27 nM. Concentrations of naturally occurring thiocyanate were below MDL for all samplings. Polythionates concentrations were determined in unfiltered samples (Figure 9) by HPLC by the same method as iodide. The MDL of this method is 0.5 µM for S_4_O_6_^2−^. Concentrations of polythionates were below MDL for all samplings. Finally, sulfite and thiosulfate concentrations were determined in unfiltered samples (Figure 9) by HPLC after derivatization with monobromobimane at room temperature in the field [120–122]. The formed derivatives were quantified by HPLC in triplicate using a C_18_ reversed-phase column (Grace, Prevail C18, 5 μm, 250 × 4.6 mm). The eluent was composed of a varying gradient of 100% methanol (eluent A) and 0.25% (v/v) acetic acid solution adjusted to pH 3.5 with 5N NaOH (eluent B) at a flow rate 1 mL min^−1^. The gradient program was as follows: start 10% A, 14 min 12% A, 30–38 min 30% A, 54 min 42% A, 82 min 80% A, 84–88 min 100% A, 90–95 min 10% A. Fluorescence (excitation at 380 nm, emission at 480 nm) was used to quantify the sulfur oxoanions. The MDL of this method is 5 nM.

### Analytical approaches to zero-valent sulfur speciation

The zero-valent sulfur pool may be defined in two ways. The first one is based on a “kinetic” approach. It includes all elemental sulfur species (e.g. rhombic cyclooctasulfur, colloidal sulfur and dissolved cyclooctasulfur) as well as inorganic polysulfides, which can release elemental sulfur at environmental conditions in seconds to minutes [91, 92]. The other approach to define the zero-valent sulfur pool is based on the presence of sulfur–sulfur bonds and includes species which are reactive toward hydrogen cyanide. The zero-valent sulfur pool as defined in this way includes polythionates and even thiosulfate in addition to all the above-mentioned species. In this work we used the “kinetic” approach, which is justified by the absence of tetrathionate in all analyzed samples.

The methods for the analysis of the zero-valent sulfur pool were critically evaluated previously [119]. The final protocol, based on the evaluation of various analytical techniques applied to rhombic and colloidal sulfur samples, as well as sulfur produced by various bacterial species, included three analytical techniques. (1) Total (sum of rhombic, colloidal, dissolved, and polysulfide) zero-valent sulfur determined by preservation of the sample with zinc acetate, followed by extraction with chloroform, and analysis of the extract by HPLC with UV–visible detector. (2) Sum of colloidal, dissolved and polysulfidic sulfur analyzed by reaction with hydrogen cyanide (cyanolysis) [46]. (3) Polysulfide sulfur concentration as calculated from the concentrations of individual polysulfide species was analyzed by fast single-phase derivatization with methyl trifluoromethanesulfonate [118]. According to the different sulfur pools extracted by these three different analytical techniques, [S^0^]_method 1_ > [S^0^]_method 2_ > [S^0^]_method 3_ [119]. On the other hand, it is known that sulfur globules produced by some bacteria are too hydrophilic to be quantitatively extracted by organic solvents [123]. The described pattern was indeed found in the water column of intertidal flat pools of the Wadden Sea [93] and in the pore-waters of Dvurechenskii mud volcano (Black Sea) [124]. In Lake Kinneret, however the concentration of zero-valent sulfur detected by cyanolysis (Method 2) was higher than that detected by the chloroform extraction (Method 1) in all horizons sampled during LK2, LK4 and LK5, as well as in some horizons during the LK3 sampling. On the contrary, in seven of the ten horizons sampled in the chemocline and hypolimnion during the LK6 sampling, the concentration of zero-valent sulfur detected by cyanolysis was lower than the one detected by chloroform extraction. LK6 sampling was performed in the last days of the lake mixing, characterized by an unstable chemocline, fast oxidation of large quantities of sulfide that resulted in the highest concentration of sulfide oxidation intermediates.

We provide two explanations for the differing results of zero-valent sulfur concentrations analysis by chloroform extraction and cyanolysis. First, the cyanolysis results may be biased to higher values by reaction of species other than elemental sulfur and polysulfides with hydrogen cyanide. Polysulfide chains with terminal organic groups may represent one type of such compounds [58, 125]. Thiosulfate also reacts with hydrogen cyanide, although this reaction is c.a. three orders of magnitude slower than the reaction between polysulfides and hydrogen cyanide [47]. Second, bacterially produced sulfur may be too hydrophilic to be extracted quantitatively with chloroform [123]. In this case, the concentration of zero-valent sulfur analyzed by chloroform extraction will be biased to lower values. The first explanation for these differences appears less likely than the second one, however, as the concentration of zero-valent sulfur was higher than the concentration of thiosulfate during all sampling periods except LK3 and the concentrations of dimethylpolysulfanes with n > 2 in Lake Kinneret are below 1 nM [58]. Further support for the incomplete chloroform extraction of zero-valent sulfur is provided by the relatively high concentrations detected during LK6 sampling, when fast mixing of sulfidic and oxic waters favors production of hydrophobic sulfur by fast chemical oxidation of sulfide. This hypothesis should be further tested by analysis of zero-valent sulfur pools using independent analytical techniques such as Cr(II) reduction [126] or voltammetry [127]. If chloroform extraction is not a quantitative method for the recovery of microbially produced elemental sulfur in the water column, the results of the sulfur isotopic analysis of elemental sulfur represent only part of its pool with bias toward elemental sulfur produced by chemical oxidation of sulfide and polysulfide sulfur. In this case, the development of analytical techniques for separation and isotopic analysis of thiocyanate produced by the cyanolysis reaction is required.

### Calculation of the depth-integrated inventories of sulfur species

In order to estimate the variations in Lake Kinneret total inventory of inorganic sulfur and its individual components, a middle Riemann sum was calculated for the sulfate, sulfide, sulfite, thiosulfate and cyanide-reactive zero-valent sulfur profiles for every sampling date. This sum is actually an estimate of the total inventory of inorganic sulfur in a water column with area of 1 m^2^, which extends from the surface of the lake to its bottom. In addition, the relative standard deviation of the total sulfur pool was calculated from the individual standard deviations of the analyses and was found to be <0.4%.

### Isotopic composition of sulfur species

**Figure 10:**
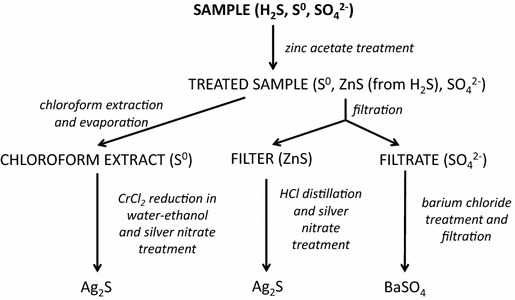
Sample processing scheme for the analysis of the isotopic composition of sulfur species.

The sample processing scheme for the analysis of the isotopic composition of sulfur species is presented in Figure 10.

For the determination of the isotopic composition of acid volatile sulfide (AVS), the samples were preserved in the field by addition of 2% (v/v) of a 200 gL^−1^ solution zinc acetate. The zinc sulfide precipitate was filtered in the laboratory on 0.45 µm nylon filters (Pall, Nylaflo). The amount of sample processed varied between 0.5 and 7.5 L according to the sulfide concentration in the samples aiming at a target amount of approximately 20 µmol sulfur in total. The filters were stored frozen in the dark. Zinc sulfide was converted to hydrogen sulfide by boiling for 2 h with 5 M HCl under a slow flow of nitrogen. The evolved hydrogen sulfide was trapped in 15 mL of silver nitrate (26 mM)—nitric acid (167 mM) solution. The silver sulfide precipitate was centrifuged, aged for at least 1 week in the dark, washed once with a 1 M ammonia solution, then three times with milli-Q water and dried overnight at 80°C.

The filtrate was used to determine the isotopic composition of sulfate. Samples for sulfate δ^34^S (δ^34^S_SO4_) and δ^18^O (δ^18^O_SO4_) were added to a 1% (v/v) 0.5 M barium chloride solution (Figure 10). The barite crystals formed were first acidified to pH 4 to dissolve any hydroxides and carbonates formed in the reaction, then centrifuged and washed 3 times with milli-Q water and dried overnight at 80°C.

Separate, unfiltered samples used to determine the isotopic composition of chloroform-extractable zero-valent sulfur were fixed in the field by addition of 2% (v/v) of a 200 gL^−1^ solution zinc acetate. Addition of zinc acetate results in the precipitation of hydrogen sulfide, thus preventing its oxidation to elemental sulfur, as well as in the decomposition of inorganic polysulfides with formation of zero-valent sulfur in the particulate form (Eq. 10) [119].

10 $$ Zn^{2 +} + S_{\text{n}}^{2 -} \to ZnS + ({\text{n}} - 1)S^{0} $$

In the laboratory, zero-valent sulfur was extracted three times with 4% v/v dichloromethane or chloroform, and the extracts were combined and dried with anhydrous calcium chloride. The dried extract was evaporated to 10–20 mL volume on a rotary evaporator. The resulting solution was evaporated to dryness in a three-neck reactor vessel under a gentle flow of nitrogen, and sulfur was reduced with CrCl_2_ in water–ethanol solution to hydrogen sulfide according to [126]. The trapping solution for hydrogen sulfide and silver sulfide purification procedure were the same as in the preparation of the AVS δ^34^S samples (Figure 10). The volume of the processed chloroform-extractable zero-valent sulfur samples varied between 3.0 and 9.5 L in order to collect enough material for analysis.

The isotope analysis of all the samples was performed in the Godwin Laboratory for Paleoclimate research at the University of Cambridge. The silver sulfide for δ^34^S_H2S_, δ^34^S_S(0)_ and barium sulfate for δ^34^S_SO4_ analysis were converted to SO_2_ at 1,030°C in a Flash Element Analyzer (EA). The sulfur isotopic composition of the resulting sulfur dioxide (SO_2_) was measured by continuous flow GS-IRMS (Thermo, Delta V Plus). Barium sulfate for δ^18^O_SO4_ was pyrolyzed at 1,450°C in a Temperature Conversion Element Analyzer (TC/EA), and the resulting carbon monoxide (CO) was measured by continuous flow GS-IRMS (Thermo, Delta V Plus). NBS 127 and IAEA SO-6 standards were used before and after every run for calibration purposes (NBS 127 δ^34^S = 20.3 ‰, δ^18^O = 8.6 ‰ and IAEA δ^34^S = −32.1 ‰), the standard deviation (1σ) ≤0.3 ‰ for all runs based on replicates of the standard. δ^34^S results are reported versus Vienna Canyon Diablo Troilite (VCDT) standard and δ^18^O results versus Vienna Standard Mean Ocean Water (VSMOW).

Error bars (±1σ) are shown for all data-points with triplicate analyses. For some data-points error bars are smaller than the symbols. Error bars for all sulfide, sulfate, and zero-valent sulfur δ^34^S data-points are 0.3 ‰ (±1σ) and represent the maximum error on the reproducibility of the standards. δ^18^O_SO4_ error bars (±1σ) are shown for all data-points with duplicate analyses and represent the standard deviation of the duplicates. For the samples which were not analyzed in duplicates, error bars represent the analytical error estimated from the analyses of standards (0.3 ‰).

### Isotope notations and calculations

In the next section we report the isotopic composition of sulfur species and sulfate oxygen in permil, using the standard delta notation:

11 $$ \delta^{34} S\left({\permil} \right) = \,\left({{{\left[{\frac{{\delta^{34} S}}{{\delta^{32} S}}} \right]_{sample}} \mathord{\left/{\vphantom {{\left[{\frac{{\delta^{34} S}}{{\delta^{32} S}}} \right]_{sample}} {\left[{\frac{{\delta^{34} S}}{{\delta^{32} S}}} \right]_{VCDT} - 1}}} \right. \kern-0pt} {\left[{\frac{{\delta^{34} S}}{{\delta^{32} S}}} \right]_{VCDT} - 1}}} \right) \cdot 1000 $$

12 $$ \delta^{18} O\left({\permil} \right) = \,\left({{{\frac{{\delta^{18} O}}{{\delta^{16} O}}_{sample}} \mathord{\left/{\vphantom {{\frac{{\delta^{18} O}}{{\delta^{16} O}}_{sample}} {\frac{{\delta^{18} O}}{{\delta^{16} O}}_{VSMOW}}}} \right. \kern-0pt} {\frac{{\delta^{18} O}}{{\delta^{16} O}}_{VSMOW}}} - 1} \right) \cdot 1000 $$

where “VCDT” stands for Vienna-Canyon Diablo Troilite and “VSMOW” stands for Vienna Standard Mean Ocean Water. We defined the isotope fractionation factor, *α*, and *ε* of the ^34^S fractionation between sulfate and sulfide as:

13 $$ {}^{34}\alpha_{B - A} = \frac{{\delta^{34} S_{B} + 1}}{{\delta^{34} S_{A} + 1}} $$

14 $$ ^{34} \varepsilon_{B - A} = \left(^{34} \alpha_{B - A} - 1\right) \times 1000 $$

We calculated *ε* for each horizon according to the following scheme. The fraction of sulfate removed by microbial sulfate reduction in the water column was calculated from concentration of sulfate at the given depth and the concentration of sulfate in the epilimnion. Then we calculated *ε* values according to criterion presented in Eq. 15. The δ^34^S values of residual sulfate and pooled sulfide were calculated according to the Rayleigh equation from the initial isotopic composition of sulfate (e.g. sulfate isotopic composition in the epilimnion) and a fraction of the sulfate reduced in the water column. This calculation is made under the assumption that microbial sulfate reduction is the only process affecting the isotopic composition of hydrogen sulfide and sulfate.

15 $$ \left(\delta^{34} S_{sulfide,\;calculated} - \delta^{34} S_{sulfate,\;calculated}\right) - \left(\delta^{34} S_{sulfide,\;measured} - \delta^{34} S_{sulfate,\;measured}\right) = 0 $$
